# Pediatric Electrocardiogram-Based Deep Learning to Predict Secundum Atrial Septal Defects

**DOI:** 10.1007/s00246-024-03540-7

**Published:** 2024-07-02

**Authors:** Joshua Mayourian, Robert Geggel, William G. La Cava, Sunil J. Ghelani, John K. Triedman

**Affiliations:** 1 Department of Cardiology, Boston Children’s Hospital, 300 Longwood Avenue, Boston, MA 02115, USA; 2 Department of Pediatrics, Harvard Medical School, Boston, MA, USA

**Keywords:** Artificial intelligence, Electrocardiogram, Atrial septal defect, Pediatric cardiology

## Abstract

Secundum atrial septal defect (ASD2) detection is often delayed, with the potential for late diagnosis complications. Recent work demonstrated artificial intelligence-enhanced ECG analysis shows promise to detect ASD2 in adults. However, its application to pediatric populations remains underexplored. In this study, we trained a convolutional neural network (AI-pECG) on paired ECG–echocardiograms (≤ 2 days apart) to detect ASD2 from patients ≤ 18 years old without major congenital heart disease. Model performance was evaluated on the first ECG–echocardiogram pair per patient for Boston Children’s Hospital internal testing and emergency department cohorts using area under the receiver operating (AUROC) and precision-recall (AUPRC) curves. The training cohort comprised of 92,377 ECG–echocardiogram pairs (46,261 patients; median age 8.2 years) with an ASD2 prevalence of 6.7%. Test groups included internal testing (12,631 patients; median age 7.4 years; 6.9% prevalence) and emergency department (2,830 patients; median age 7.5 years; 4.9% prevalence) cohorts. Model performance was higher in the internal test (AUROC 0.84, AUPRC 0.46) cohort than the emergency department cohort (AUROC 0.80, AUPRC 0.30). In both cohorts, AI-pECG outperformed ECG findings of incomplete right bundle branch block. Model explainability analyses suggest high-risk limb lead features include greater amplitude P waves (suggestive of right atrial enlargement) and V1 RSR’ (suggestive of RBBB). Our findings demonstrate the promise of AI-pECG to inexpensively screen and/or detect ASD2 in pediatric patients. Future multicenter validation and prospective trials to inform clinical decision making are warranted.

## Introduction

Secundum atrial septal defect (ASD2) is a common congenital heart defect. ASD2 detection is often delayed, making it the most frequent congenital heart lesion initially diagnosed in adults [1]. Late diagnosis complications include atrial tachyarrhythmias, right ventricular dysfunction, pulmonary hypertension, and paradoxical embolus [2–4], underscoring the need for early detection/intervention. The challenge of diagnosing ASD2 in pediatric populations is attributed to subtle physical exam features (e.g., wide fixed split S2) and absence of symptoms in early life [1]. While ECG is conventionally considered an insensitive screening tool for ASD2 detection [1, 5, 6], recent work demonstrated artificial intelligence-enhanced ECG (AI-ECG) analysis shows promise to detect ASD2 in adults [7]. However, model performance for ASD2 < 10 mm (representative of the majority of pediatric ASD2 cases [8]) was limited with an area under the receiver operating curve (AUROC) of 0.65; in addition, the patterns of normal versus abnormal pediatric ECGs differ significantly from adults, which may limit the application of adult AI-ECG algorithms to pediatric cohorts [9]. Altogether, this underscores the need for a pediatric-specific model.

In this study, we aim to address this gap by training and testing a convolutional neural network (AI-pECG) on paired ECG–echocardiograms to detect ASD2 from patients ≤ 18 years old without major congenital heart disease.

## Methods

### Internal Study Population and Patient Assignment

The internal study cohort and patient assignment is detailed elsewhere [10]. Briefly, patients ≤ 18 years old without major congenital heart disease based on institutional Fyler codes were included (note patent foramen ovale is included, whereas primum ASD is excluded). Only the closest ECG–echocardiogram pair ≤ 2 days apart was included. ECGs failing to pass quality control were removed, with the remaining data comprising the main cohort.

A group stratified design was implemented as previously described [10] to partition the main cohort, restricting ECG–echocardiogram pairs for a given patient to either training or testing cohorts.

### Data Retrieval, Quality Control, and Data Preprocessing

Data retrieval, quality control, and data preprocessing are detailed elsewhere [10]. Briefly, raw ECG waveforms were obtained from an internal database, where each one-dimensional vector of lead data was sampled at a rate of 250 Hz for 10 s of duration (2500 samples). An ECG was discarded if any lead is not 2500 samples long, or if any lead recording has no lead information (i.e., flat line). A high pass filter was utilized [11] to remove baseline wander, followed by trimming to 2048 samples (approximately 8 s) to facilitate conveniently working with convolution neural networks.

In addition, the following data were retrieved in this study: (1) diagnoses of ASD2 based on institutional Fyler codes; (2) pediatric cardiologist expert ECG-based diagnosis of incomplete right bundle branch block (IRBBB).

### Definition of Primary Outcomes

The primary outcome, ASD2, was classified using echocardiogram-based institutional Fyler codes.

### Model Selection, Architecture, and Training

The model was developed solely on the training set. Model selection, architecture, and training are detailed elsewhere [10], with final hyperparameters after tuning of kernel size 17, batch size 32, and learning rate 0.001.

### Performance Evaluation and Statistical Analyses

Model performance was evaluated on the first ECG–echocardiogram pair per patient for Boston Children’s Hospital internal testing and emergency department cohorts using area under the receiver operating (AUROC) and precision-recall (AUPRC) curves. Sensitivity and positive predictive value (PPV) were calculated using two different thresholds achieving the following in the training cohort: A) 75% PPV and B) 95% sensitivity. For benchmarking purposes, performance was compared to pediatric cardiologist expert ECG-based diagnosis of incomplete right bundle branch block.

### Model Explainability

The following model explainability analyses were performed as previously described [10]: 1) median waveform analysis and 2) saliency mapping. Briefly, median waveform analysis generates a representative low- and high-risk ECG median waveform using the 100 lowest and 100 highest predicted ECGs to have an ASD2, respectively. Saliency mapping highlights regions of the ECG most influential in model predictions. Saliency was averaged over the 100 highest predicted ECGs of the primary outcome.

### Data Availability and Software

Requests for Boston Children’s Hospital data and related materials will be internally reviewed to clarify if the request is subject to intellectual property or confidentiality constraints. Shareable data and materials will be released under a material transfer agreement for non-commercial research purposes. Use of Boston Children’s Hospital data was approved by its Institutional Review Board.

Programming code used to perform the analyses is available upon reasonable request. The convolutional neural network used the Keras framework with a Tensorflow (Google) backend using Python 3.9 [12]. Deep learning was executed on institutional graphics processing units. All other pre- and post-processing codes were written in Python 3.9 [12] and R 4.0 [13], which were executed locally.

## Results

### Patient Population Characteristics

The training cohort comprised of 92,377 ECG–echocardiogram pairs (46,261 patients; median age 8.2 [IQR, 2.9–13.8] years) with an ASD2 prevalence of 6.7%. Test groups—which utilized the first ECG–echocardiogram pair per patient—included internal testing (12,631 patients; median age 7.4 [IQR, 1.5–13.7] years; 14.6% with IRBBB; 6.9% ASD2 prevalence) and emergency department (2,830 patients; median age 7.5 [IQR, 1.2–14.5] years; 11.4% with IRBBB; 4.9% ASD2 prevalence) cohorts. Other demographic details have been previously published for this cohort [10].

### AI-pECG Model Performance

**Fig. 1:**
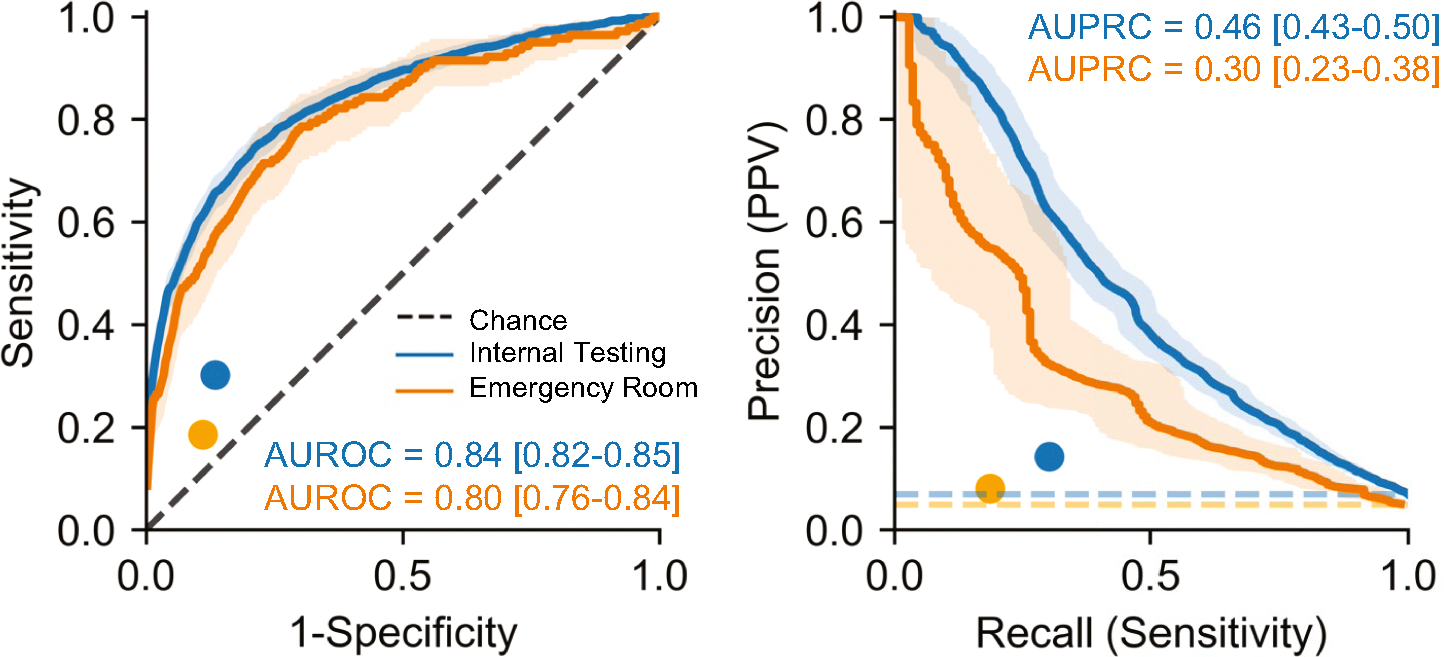
Pediatric electrocardiogram-based deep learning to predict atrial septal defects. Performance of the artificial intelligence-enhanced pediatric electrocardiogram model performance evaluated using the internal test (blue) and emergency department (orange) cohorts with receiver operating (AUROC; left) and precision-recall (AUPRC; right) curves. Color-coded dots represent the benchmark of pediatric cardiologist expert ECG-based diagnosis of incomplete right bundle branch block. AUROC and AUPRC metric values for each model and outcome are inset. Dotted line represents chance. 95% confidence intervals are shown using bootstrapping. Abbreviations: positive predictive value (PPV) (Color figure online)

After training the AI-pECG model on nearly 100,000 ECG–echocardiogram pairs with corresponding human expert detection of ASD2 on echocardiogram, model performance was evaluated. Model performance was higher in the internal test (AUROC 0.84, AUPRC 0.46) cohort than the emergency department cohorts (AUROC 0.80, AUPRC 0.30) (Fig. 1). In both cohorts, AI-pECG outperformed ECG findings of IRBBB, which had a sensitivity of 30.1%, specificity of 86.5%, and PPV of 14.2% in the internal test cohort, and sensitivity of 18.6%, specificity of 89.0%, and PPV of 8.1% in the emergency department cohort (Fig. 1).

At threshold A (0.47), a PPV of 72% and 57% was achieved with sensitivities of 25% and 15% in test and emergency department cohorts, respectively (Table 1). At threshold B (0.04), PPVs of 13% and 9% and sensitivities of 88% and 84% were achieved, respectively (Table 1).

### Subgroup Analysis

**Fig. 2:**
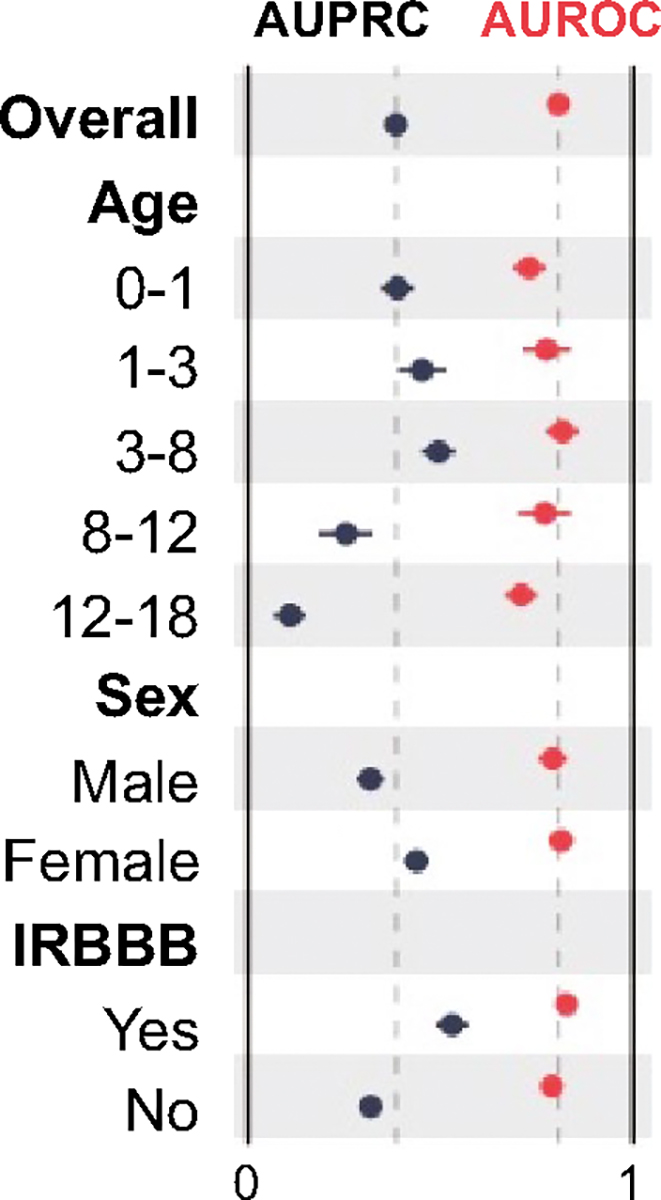
Subgroup Model Performance. Forest plot showing AUROC (red) and AUPRC (black) when stratifying by age (in years), sex, and incomplete right bundle branch block (IRBBB). 95% confidence intervals are shown using bootstrapping (Color figure online)

As shown in Fig. 2, model performance was higher for predicting ASD2 for ages < 8 years old, females, and ECG findings of IRBBB.

### Model Explainability

**Fig. 3:**
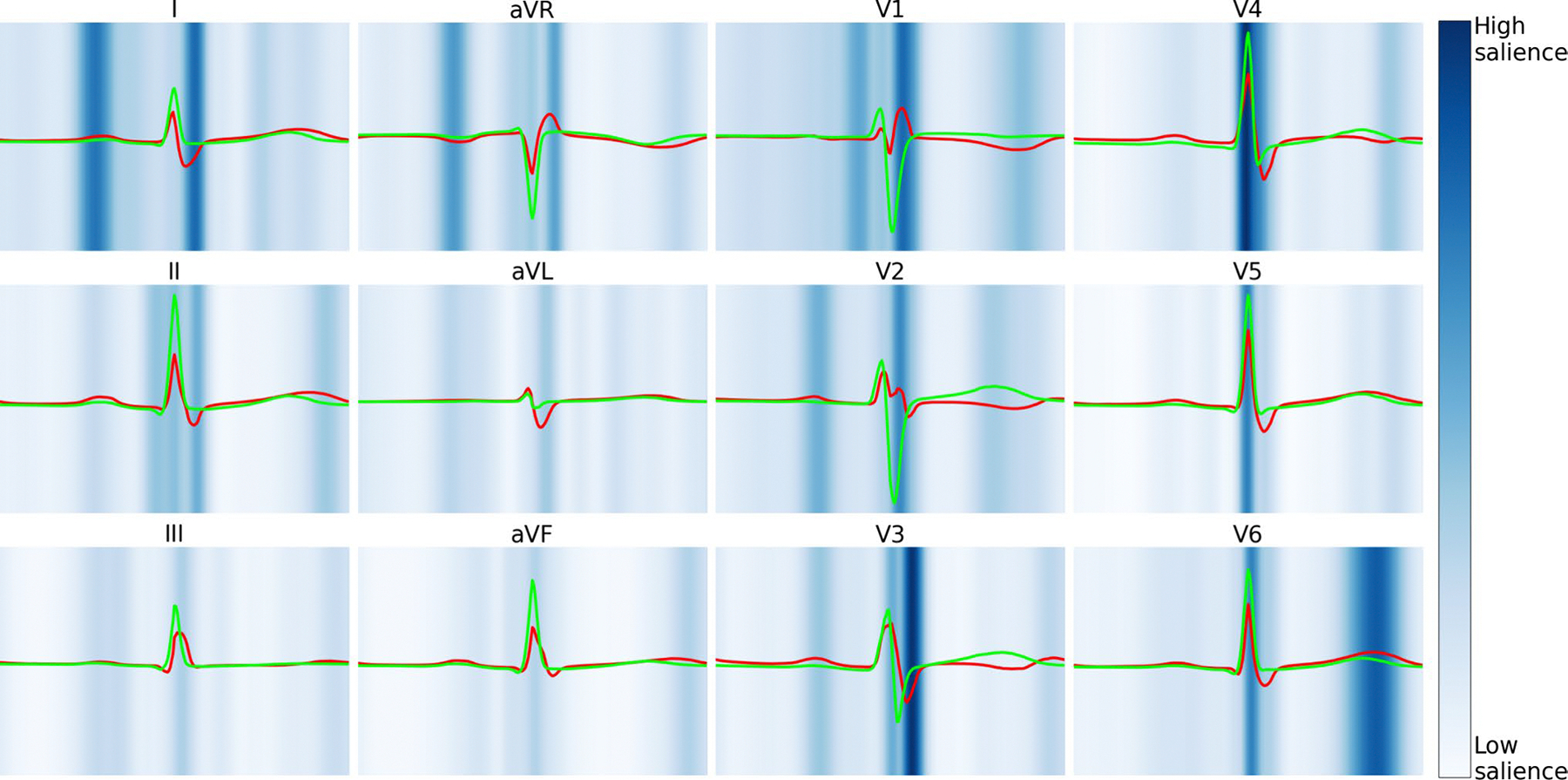
Explainability of AI-pECG Predictions. Visualization of median waveforms generated in each lead using ECGs from the 100 highest (red) and 100 lowest (green) AI-pECG predictions. Saliency mapping demarcates regions of the ECG waveform having greatest (dark blue) and least (light blue) influence on each outcome. Saliency was averaged over the 100 highest predicted ECGs of the primary outcome (Color figure online)

Finally, in an attempt to gain model interpretability, saliency mapping and median waveform analysis were performed (Fig. 3).

Salient limb lead (I and aVR) features to predict ASD2 include P and S waves. In the precordial leads (V1-V6), salient features include the QRS complexes and T waves. High-risk limb lead (I and aVR) features include greater amplitude P waves suggestive of right atrial enlargement. High-risk V1 precordial lead features include RSR’ suggestive of RBBB, accompanied by V3-V6 prolonged QRS interval. High-risk V6 precordial lead features include a higher-amplitude T wave.

## Discussion

In this work, a technological gap was addressed by applying ECG-based deep learning to a pediatric cohort for prediction of ASD2. After training a model on nearly 100,000 ECG–echocardiogram pairs ≤ 2 days apart, performance was tested on > 10,000 patients from an independent internal test cohort, as well as nearly 3,000 patients from a separate clinical setting (emergency department) at Boston Children’s Hospital. Finally, saliency mapping was performed to provide model explainability and identify regions of the ECG waveform that influence model predictions.

### Conventional ECG Findings to Detect ASD2

Previous work has attempted to utilize conventional rule-based ECG analysis to detect pediatric ASD2 with modest performance [1, 5, 6]. Similar to our study, Schiller et al. had a prevalence of ASD2 of 7.1% and IRBBB of 17.9% [5]. In addition, they found that ECG IRBBB sensitivity was low for diagnosing ASD2 (36.1%), with a specificity of 80% and PPV of 14.7%. These numbers closely resemble our findings herein (e.g., sensitivity of 30.1%, specificity of 86.5%, and PPV of 14.2% on the internal testing cohort). Earlier work had also assessed the utility of right ventricular enlargement, with limited model performance [6]. Together, this underscores the need for a novel tool to detect ASD2.

### Comparison of Model Performance to Previous Literature

As shown in Fig. 1, our model outperformed IRBBB known to have limited sensitivity and positive predictive value in children [5] and adults [7]. AUROCs and AUPRCs were slightly lower than the adult counterpart model [7] in the setting of subtle ASD2-related ECG changes in younger patients (in a recent small single-center study, only adult patients with ASD2 were found to have more striking ECG changes such as complete RBBB, atrial fibrillation, or atrial flutter [1]). Of note, our overall model performance was higher than the adult counterpart model’s performance for ASD2 < 10 mm (AUROC of 0.65) [7].

### Comparison of Internal Testing and Emergency Department Cohorts

As shown in Fig. 1, there was a slight decrease in performance between the internal testing and emergency department cohort. We hypothesize this may be related to inherent differences in the clinical settings, as the emergency department is likely to have higher acuity/illness that may be reflected in the ECGs. For example, the heart rate in the emergency department cohort was significantly higher than the testing cohort (112 [IQR 87–140] vs. 90 [IQR 73–120] beats per minute, respectively; p < 0.001) despite similar ages. In addition, the internal testing cohort includes cardiology clinic, where the index of suspicion for ASD2 may be higher (which may lead to a more focused echocardiographic assessment of the atrial septum), whereas the emergency department is likely to have higher acuity (where the echocardiogram indication may deprioritize atrial septum assessment). Finally, we acknowledge that the clinical setting of the training cohort is identical to the internal testing cohort, whereas the emergency department setting was excluded from training.

### Clinical Value of AI-pECG

Our select thresholds may reasonably be expected to A) prompt cardiology referral and facilitate earlier and more frequent detection of ASD2 (threshold A) or B) help rule out ASD2 (threshold B). Table 1 suggests when using threshold A, 15–25% of ASD2 would be captured at a PPV of 57–72%. At threshold B, approximately 53% of ECGs would help rule out ASD2 at a sensitivity of 84–88%. This would therefore have potential to decrease echocardiograms for ASD2 screening indications by 53%.

### Insight Gained from AI-pECG

From a model explainability perspective, model performance was higher in ages < 8 years old, suggesting the model is capturing native electrophysiologic changes independent of progressive disease burden. Saliency mapping and median waveform analysis reinforce conventional ECG findings suggestive of ASD2 (e.g., RBBB, right atrial enlargement), and provide insight into novel ECG markers.

### Limitations and Future Directions

We acknowledge several limitations of this work. First, while the model performs well across multiple clinical settings internally, true external validation is warranted. Second, only two example thresholds were used to detect disease. Further consideration is required to weigh the impact of resultant false positives (which may lead to unnecessary referrals to echocardiogram) and false negatives (which may lead to clinical consequences of missed ASD2). Third, our database does not facilitate investigation into model performance by ASD2 size. Finally, while saliency mapping provides insight into model behavior, its limitations must be noted [14].

## Conclusions

In conclusion, our findings demonstrate the promise of AI-pECG to inexpensively screen and diagnose ASD2 in pediatric patients, which may serve as a potential cost saving tool to avoid unnecessary echocardiograms. This tool may facilitate prioritization of patients for future interventions/studies and provide meaningful insight into novel ECG waveforms suggestive of ASD2. Future multicenter validation and prospective trials to inform clinical decision making are warranted.

## Figures and Tables

**Table 1:** Summary of model performance at select thresholds

	Boston Children’s Hospital	
	Internal testing	Emergency department

*Threshold A = 0.47*		
Prevalence (%)	6.9	4.9
Sensitivity	0.25 [0.22–0.28]	0.15 [0.09–0.21]
Specificity	0.99 [0.99–0.99]	0.99 [0.99–1.00]
NPV (%)	94.7 [94.5–94.9]	95.7 [95.5–96.1]
PPV (%)	72.0 [67.3–76.6]	57.4 [41.2–73.7]
Predicted negative (%)	97.6 [97.4–97.9]	98.7 [98.3–99.1]
*Threshold B = 0.04*		
Prevalence (%)	6.9	4.9
Sensitivity	87.6 [85.3–89.6]	84.4 [77.9–90.0]
Specificity	55.8 [54.9–56.7]	55.2 [53.3–57.0]
NPV (%)	98.4 [98.1–98.6]	98.6 [98.0–99.0]
PPV (%)	12.8 [12.5–13.2]	9.0 [8.3–9.6]
Predicted negative (%)	52.8 [52.0–53.7]	53.3 [51.5–55.1]

Data presented as median [95% confidence interval]. Predicted Negative indicates the fraction of ECGs predicting negative echocardiogram findings at the given threshold

*NPV* negative predictive value, *PPV* positive predictive value
